# Electrochemical DNA Sensor Based on Poly(Azure A) Obtained from the Buffer Saturated with Chloroform

**DOI:** 10.3390/s21092949

**Published:** 2021-04-22

**Authors:** Anna Porfireva, Kseniya Plastinina, Vladimir Evtugyn, Yurii Kuzin, Gennady Evtugyn

**Affiliations:** 1 A.M. Butlerov’ Chemistry Institute of Kazan Federal University, 18 Kremlevskaya Street, 420008 Kazan, Russia; Anna.Porfireva@kpfu.ru (A.P.); plastininak@bk.ru (K.P.); Yurii.Kuzin@kpfu.ru (Y.K.); 2 Interdisciplinary Center of Analytical Microscopy of Kazan Federal University, 18 Kremlevskaya Street, 420008 Kazan, Russia; Vladimir.Evtugyn@kpfu.ru; 3 Analytical Chemistry Department of Chemical Technology Institute of Ural Federal University, 19 Mira Street, 620002 Ekaterinburg, Russia

**Keywords:** Azure A, electropolymerization, electrochemical DNA sensor, electrochemical impedance spectroscopy, DNA damage detection

## Abstract

Electropolymerized redox polymers offer broad opportunities in detection of biospecific interactions of DNA. In this work, Azure A was electrochemically polymerized by multiple cycling of the potential in phosphate buffer saturated with chloroform and applied for discrimination of the DNA damage. The influence of organic solvent on electrochemical properties of the coating was quantified and conditions for implementation of DNA in the growing polymer film were assessed using cyclic voltammetry, quartz crystal microbalance, and electrochemical impedance spectroscopy. As shown, both chloroform and DNA affected the morphology of the polymer surface and electropolymerization efficiency. The electrochemical DNA sensor developed made it possible to distinguish native and thermally and chemically damaged DNA by changes in the charge transfer resistance and capacitance.

## 1. Introduction

There is an urgent need in the development of simple and reliable analytical devices for the detection of antioxidants, drugs, biomarkers, and toxic species required in medical diagnostics and food safety assessment [1,2,3,4,5]. Although conventional instrumentation, e.g., high performance liquid chromatography and capillary electrophoresis, offer quite sensitive and selective analysis of most analytes mentioned, they are rather expensive, time- and labor-consuming, and cannot provide necessary information in point-of-care (POC) format [6,7]. Immunoassay techniques frequently used in hospitals have some limitations related to the insufficient stability of antibodies and enzymes used as labels in the ELISA protocols and to interfering matrix effects, especially in testing biological liquids [8]. From other sensors utilized in determination of biologically active low molecular compounds, DNA-based assay is considered as one of most promising due to high variety of the species detected, higher stability of DNA oligonucleotides and aptamers against proteins, and simpler detection mode [9,10]. In particular, DNA-based sensors have been described for sensitive determination of antitumor drugs [11,12,13,14], reactive oxygen species [15], and antioxidants [16,17,18]. Aptamer-based biosensors detect with very high sensitivity mycotoxins, drugs, and cancer biomarkers [19,20,21,22,23,24,25]. Meanwhile, the detection of the DNA (aptamer)-analyte interactions and its conversion into an analytical signal remain a weak point of such biosensors. Traditional approaches require introduction of specific labels and are time consuming, although the sensitivity of the analyte determination is quite acceptable. Label-free biosensors utilize changes in permeability of the sensing layers caused by analyte implementation and measured mostly by electrochemical impedance spectroscopy (EIS) [26,27]. However, unspecific adsorption of the sample components affects the response of such biosensors, especially those based on Au able to form bonds with biogenic thiols. Necessity in additional treatment of the surface to block naked parts of the transducer not only complicates the biosensor assembling, but also decreases absolute values of the signal recorded.

Redox active polymers are frequently used in the development of label-free DNA sensors [28,29]. Polymers, mostly obtained by electropolymerization, show high efficiency of the DNA immobilization via electrostatic interactions and suppress undesired interactions with interferences. Then, noncovalent interactions between the underlying redox active support and biopolymer result in the changes of both electrostatic interactions and permeability of the surface layer. Interaction with analyte molecules affects both parameters due to partial shielding of the charges and/or charge separation in the recognition event. For this reason, monitoring of the redox activity of the polymers shows high sensitivity toward specific DNA interactions. Thus, DNA sensors based on polyaniline [30,31,32,33,34], polypyrrole [35,36,37], polythionine [38], and poly(neutral red) [15,39] have been described and successfully used for the determination of intercalation, DNA damage, and detection of hybridization with complementary DNA sequences. It should be noted that application of such modifiers frequently limits the measurement conditions by pH region required for redox signal recording (polyaniline) or by interference with reactive species able to react with electrodes near the redox activity of appropriate polymers (polyphenothiazines). Rather low selectivity of DNA intercalation monitoring was also reported [12,40].

Recently, we showed that polymerization of Azure B and proflavine followed by adsorption of double-stranded DNA results in sensitive determination of anthracycline drugs [40,41]. However, low solubility and aggregation of the dyes in solution and on the electrode surface can affect their polymerization and sensitivity of their interaction with DNA molecules. Meanwhile, electropolymerization from organic solvents seems less appropriate for the DNA biosensors due to hydrophobicity of the polymer interphase and difficulties in its compatibility with highly polar DNA molecules. Films polymerized from organic solvents are normally denser than those deposited from aqueous solutions and contain less water and small ions. This complicates charge transfer and redox signal generation on the polymer–DNA interface. In this work, we propose to perform electropolymerization in the presence of chloroform to improve the performance of the DNA sensor and increase sensitivity of the signal toward anthracycline drugs. Low amounts of organic solvent improve electropolymerization conditions due to higher solubility of the monomer while not affecting implementation of DNA molecules in the growing polymer film.

## 2. Materials and Methods

### 2.1. Reagents

Azure A (dye content 80%), DNA from fish sperm, and chloroform were purchased from Sigma (https://www.sigmaaldrich.com/ accessed on 11 January 2021). All the working solutions were prepared using Millipore Q^®^ water. Electrochemical measurements were performed in 0.1 M phosphate buffer containing 0.1 M NaNO_3_ (pH 7.0). In the pH dependence experiments, appropriate pH value was adjusted in the range from pH = 2.0 to 8.0 by adding 0.1 M HCl or NaOH. Saturation of the working buffer with chloroform was performed by mixing in 4:1 volume ratio and stirring for 30 min. After phase separation, the aqueous part was taken for the electropolymerization experiments.

### 2.2. Apparatus

Voltammetric measurements were performed at ambient temperature using a portable bipotentiostat-galvanostat μStat 400 Metrohm DropSens (DropSens, S.L., Asturias Llanera, Spain). EIS measurements were performed with the FRA 2 module of the potentiostat-galvanostat AUTOLAB PGSTAT 302N (Metrohm Autolab b.v., Utrecht, Netherlands).

Three-electrode cells equipped with the glassy carbon electrode (GCE, 0.0167 cm^2^) Pt stripe as auxiliary electrode and Ag/AgCl/3 M KCl reference electrode was used for cyclic voltammetry (CV) and EIS measurements. In the EIS experiments, the potential frequency was varied from 100 kHz to 0.04 Hz, amplitude of the applied sine potential was equal to 5 mV, and equilibrium potential was calculated as a half-sum of the peak potentials recorded in a 0.01 M [Fe(CN)_6_]^3−/4−^ pair as the redox probe. Measurements were performed in 0.1 M phosphate buffer containing 0.1 M NaNO_3_. The impedance parameters were calculated from the Nyquist diagram corresponding to the *R*(*RC*)(*RC*) equivalent circuit using NOVA software (Metrohm Autolab b.v., Utrecht, Netherlands).

Electrochemical quartz crystal microbalance (EQCM) measurements were performed with the EQCM module of the CHI 440B instrument (CH Instruments, Inc. Austin, Texas, USA) equipped with the EQCM chip (basic frequency 8 MHz, 0.205 cm^2^) and Au thin-film electrodes. 

Scanning electron microscopy (SEM) images of the electrode coatings were obtained with a Merlin™ (Carl Zeiss AG, Oberkochen, Germany) high-resolution field emission scanning electron microscope.

Atomic force spectroscopy (AFM) images of glassy carbon sheets covered with electropolymerized films were obtained with a Dimension FastScan (Bruker, Karlsruhe, Germany) scanning probe microscope in the mode of quantitative nanomechanical mapping using silicon probes “Bruker scanasyst air” (curvature radius ~2 nm) and *k* 0.4 N/m. Scan rate was equal to 1 Hz within a 256 × 256 window. Image processing was performed with the Gwyddion–Free SPM data analysis software.

### 2.3. Azure A Electropolymerization and DNA Sensor Assembling

Prior to the Azure A electropolymerization, the GCE electrode was mechanically polished and cleaned with acetone and deionized water. Next, it was electrochemically cleaned by repeated potential cycling in 0.1 M H_2_SO_4_ until the background current stabilized. After that, it was immersed in 5 mL of the working buffer containing 0.2 mM Azure A. In some experiments, phosphate buffer was saturated with chloroform, as described above. The potential of the electrode was multiply cycled between −0.6 and 1.2 V with a scan rate of 100 mV/s. The electrode was then washed with deionized water and working buffer and dried in air at ambient temperature for 20 min. Codeposition of poly(Azure A) and DNA was performed in a similar manner. DNA was added to the Azure A dissolved in phosphate buffer to its final concentration of 0.2 mg/mL. Native, thermally denatured, and oxidized DNA samples were tested in these experiments. Thermal denaturation was performed by heating the DNA stock solution for 30 min at 95 °C followed by sharp cooling in crushed ice for 5 min. Oxidatively damaged DNA was performed by its treatment with the mixture of 0.9 mL of 4 mM CuSO_4_ and 1.3 μL of 30% H_2_O_2_ for 1 h.

## 3. Results

### 3.1. Electropolymerization of Azure A from Its Aqueous Solution

**Figure 1:**
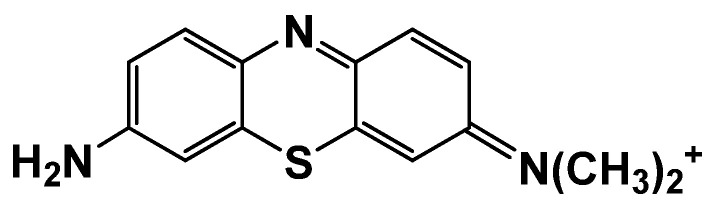
Chemical structure of Azure A.

Azure A is a phenothiazine dye with a sterically hindered amino group from one side of the phenothiazine core and primary amino group from the opposite side (Figure 1).

**Figure 2:**
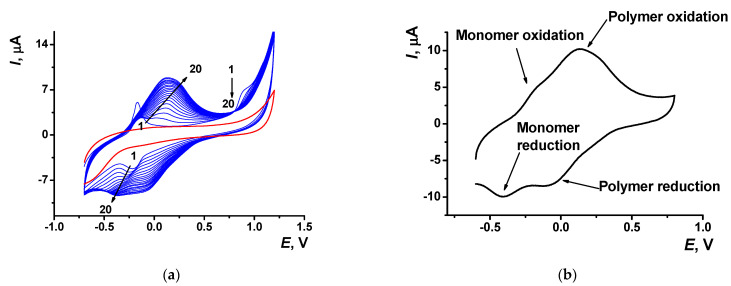
(**a**) Multiple cyclic voltammograms recorded on the GCE in 0.1 M phosphate buffer containing 0.1 M NaNO_3_ and 0.2 mM Azure A, pH = 7.0; scan rate 100 mV/s. Red line corresponds to the bare GCE. Arrows indicate changes in the peaks from the 1st to 20th cycle. (**b**) Single cycle recorded on the GCE covered with poly(Azure A) transferred in the same buffer with no monomer dye.

Electropolymerization of Azure A resulted in specific changes of the peaks on cyclic voltammograms (Figure 2) attributed to the deposition of redox active product on the electrode surface. At first scan, a pair of reversible redox peaks related to the redox conversion of the monomer appeared at about −0.17 and −0.22 V. In addition, an irreversible oxidation peak of the cation radical formation was found at high anodic potentials (about 0.9 V). In the following cycle of the potential, a new pair of the redox peaks shifted to higher anodic potentials (−0.13 and 0.14 V) against monomer peaks appeared and started growing (Figure 2a).

Transferred to the working buffer with no monomer, the GCE modified with the poly(Azure A) demonstrated two pairs of the peaks on the cyclic voltammogram (Figure 2b). For structurally relative Azure B, similar peaks were referred to the polymer and the monomer entrapped in the growing polymer film [42]. Stability of the redox signals was assessed for six electrodes modified by means of the same set of reagents. In ten consecutive potential scans, changes in the redox peaks related to the poly(Azure A) were negligible, whereas the oxidation peak current of the monomeric dye decreased by 20% and that of the reduction peak current by 45%.

The slope of the bilogarithmic dependence of the peak current (*I_p_*) on the scan rate (ν) indicated diffusion control of the monomer oxidation (d(log*I_p_*)/d(logν) = 0.44 ± 0.02) and mixed diffusion–adsorption control of the polymer conversion (0.84 ± 0.03 for oxidation peak current and 0.81 ± 0.03 for reduction peak current).

Variation of the pH influenced the signals of monomeric and polymeric forms of Azure A in a different manner (Figure S1, Supplementary Materials). Thus, oxidation peak current of the monomeric dye was stable in acidic media and decreased when transferred in the neutral and alkaline media. Oxidation peak current of the polymeric form regularly decreased in the pH range from 3.0 to 7.0. In alkaline solution, the signal became higher. The difference in the behavior of the monomer and polymer observed could be attributed to the alternative reaction of the dyes with dissolved oxygen and pH-dependent accessibility of the redox centers of the layer components toward this oxidant. The reduction peak current of the monomeric dye increased with pH in acidic and neutral media and reached a flat maximum at pH > 7.0. The appropriate peak of the polymeric form was pH independent.

Alternative chemical oxidation of reduced Azure A fragments by dissolved oxygen was confirmed by appropriate experiments performed after oxygen removal by the nitrogen (Figure S2, Supplementary Materials). Both anodic and cathodic peak currents increased against those recorded in the presence of oxygen. The effect is more pronounced in acidic media where the current shift was about 40%, while in neutral and basic media it was about 25% and did not significantly depend on the pH value. The shape and position of the peaks remained the same after the oxygen removal. Together with reproducibility of the peaks in a series of experiments performed with the same sensor, this means the nature of the products obtained in chemical and electrochemical oxidation is the same.

**Figure 3:**
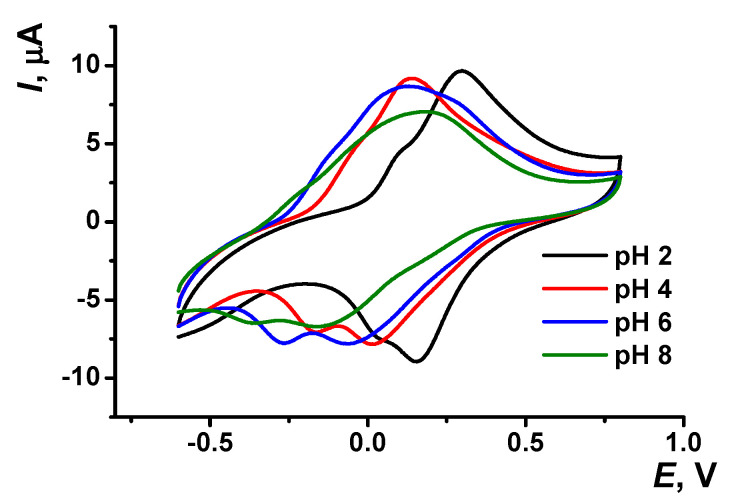
Cyclic voltammogram recorded on the GCE modified with poly(Azure A) in 0.1 M phosphate buffer at different pH values; scan rate 100 mV/s.

The pH dependency of voltammograms obtained with the GCE covered with poly(Azure A) in the working buffer with no monomer is illustrated in Figure 3. The peak currents are insignificantly decreasing with the pH increase. The effect is more pronounced in basic media and for cathodic peaks. Changes observed can be attributed to the influence of protonation–deprotonation within the polymer film on the electron exchange between reduced and oxidized forms of the dye, both in monomeric and polymeric forms.

**Figure 4:**
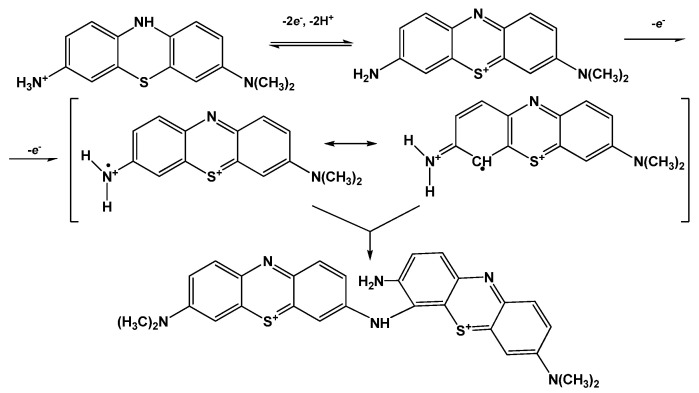
Mechanism of Azure A electropolymerization.

Both peak potentials of the polymer in acidic media (pH = 2.0–6.0) and anodic peak potential of the monomer depended linearly on the pH with the slope close to −59 mV/pH indicating equal number of the hydrogen ions and electrons transferred. This coincides well with the mechanism of Azure A electropolymerization proposed elsewhere for similar conditions of potential scanning [43]. An appropriate reaction scheme is presented in Figure 4. It involves the formation of cation radicals followed by their coupling and stabilization by hydrogen ion release.

The pH dependence of the reduction peak of the monomer showed a slope of −73 mV/pH. This can be related to the pH-dependent alternative reaction of the monomer oxidation with dissolved oxygen or pH-caused changes in the accessibility of the monomeric dye molecules toward electron exchange. It should be noted that the pH influence was quite reversible and appropriate peak positions could be restored by the opposite pH shift in the range studied (pH = 2.0–8.0). At higher pH, the polymer film becomes chemically instable, probably due to oxidative decomposition, so that the peaks on the voltammograms changed irregularly within the pH range studied.

### 3.2. Electropolymerization of Azure A in the Buffer Saturated with Chloroform

**Figure 5:**
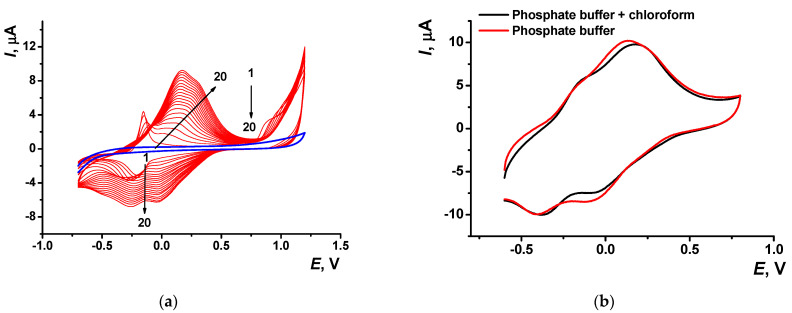
(**a**) Multiple cyclic voltammograms recorded on the GCE in 0.1 M phosphate buffer containing 0.1 M NaNO_3_ and 0.2 mM Azure A and saturated with chloroform, pH = 7.0; scan rate 100 mV/s. Arrows indicate changes in the peaks from the 1st to 20th cycle, blue line corresponds to bare GCE. (**b**) Single cycle recorded on the GCE covered with poly(Azure A) transferred in the same buffer with no monomer dye.

Deposition of the poly(Azure A) film from the phosphate buffer saturated with chloroform (denoted below as poly(Azyre A)/Chl) showed similar peaks on the voltammogram. The peak pairs related to the monomer were shifted to less cathodic potentials (−0.15 and −0.20 V) against those previously described in phosphate buffer with no solvent. Cathodic peaks on the reversed branch of cyclic voltammograms were much better resolved (Figure 5). Shape of the same peaks recorded in the absence of the monomer (Figure 5b) was less sensitive to the presence of organic solvent, assuming similar mechanism of the electrode reactions. Meanwhile, the stability of the polymeric film obtained in the presence of chloroform was higher than that of the film deposited in conventional conditions. The reduction peak of the polymer was stable for at least 10 consecutive scans of the potential, whereas anodic peak tended to decrease in the same series by 15%.

In accordance with the dependence of the peak currents on the scan rate, redox conversion of the monomeric dye was controlled by diffusion (d(log*I_p_*)/d(logν) = 0.53 ± 0.01 and 0.40 ± 0.02 for oxidation and reduction, respectively) whereas polymeric dye showed mixed (adsorption–diffusion) control (d(log*I_p_*)/d(logν) = 0.80 ± 0.01 and 0.85 ± 0.02).

The pH dependence of the peak parameters on voltammograms recorded in the Azure A solution saturated with chloroform are presented in Figure S3, Supplementary Materials. In comparison with the peaks recorded in absence of organic solvent, monomer peak currents were higher and those of the polymer coatings slightly lower.

**Figure 6:**
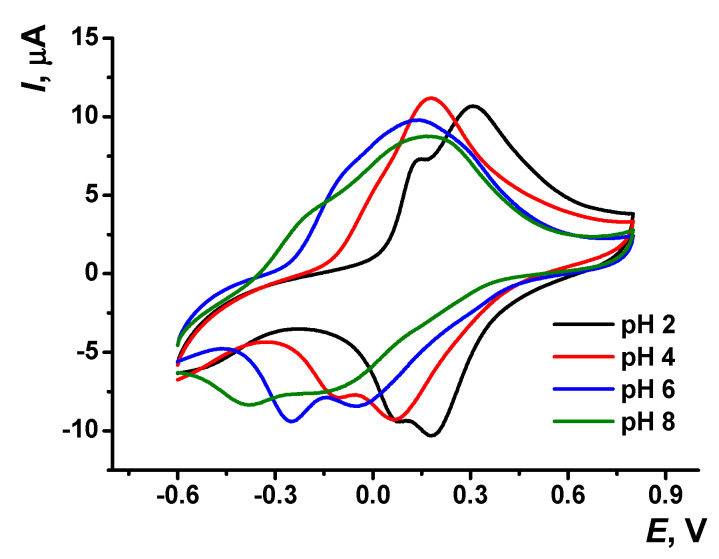
Cyclic voltammograms recorded on GCE covered with poly(Azure A/Chl), 0.1 M phosphate buffer containing 0.1 M NaNO_3_; scan rate 100 mV/s.

Cyclic voltammograms recorded on the GCE modified with poly(Azure A)/Chl in the absence of the monomeric dye in solution are presented in Figure 6. As in the case of poly(Azure A), the peaks of the poly(Azure A)/Chl shifted to more cathodic potentials with increasing pH values. The parameters of the *Ep*–pH dependencies are summarized in Table S1. The slope of the *Ep*–pH dependence mostly corresponded to the equal number of the H^+^ ions and electrons transferred (−57 ± 2 mV/pH for the monomer oxidation, −60 ± 0.4 mV/pH and −58 ± 0.3 mV/pH for the polymer oxidation and reduction). The slope of the pH dependence of the monomer reduction peak was significantly higher (−83 ± 0.3 mV/pH). The linearity of appropriate dependencies was broader than that of the poly(Azure A) and covered the range of pH = 2.0–7.0.

### 3.3. DNA Implementation in Surface Layers

Implementation of DNA into the redox active polymer layer is commonly performed by drop casting of the DNA solution [40] or addition of the DNA to the reaction media on the electropolymerization stage [14,44]. Adsorption of native DNA added to the monomer solution within the electropolymerization process led to the decrease of the currents on voltammograms. Thermal denaturing and chemical oxidation of the DNA molecules with reactive oxygen species further suppressed the currents recorded. Typical voltammograms are presented in Figure S3, Supplementary Materials.

#### 3.3.1. EQCM Measurements

The influence of the DNA addition to the Azure A electropolymerization was confirmed by EQCM measurements. The frequency of the quartz oscillation depends on the mass deposited onto the electrode surface, in accordance with Sauerbrey Equation (1) [45].

(1) $$\Delta f=-\frac{2f_{0}^{2}\Delta m}{A\sqrt{\rho_{q}\mu_{q}}}=-C_{f}\Delta m$$

where Δ*f* is the shift of the quartz resonance frequency, *A* the area of Au electrodes, *ρ_q_* the quartz density, *μ_q_* shear modulus, *f*_0_ fundamental resonance frequency of the quartz crystal, *C_f_* the sensitivity coefficient, and Δ*m* the surface mass change. Although Equation (1) describes the deposition of the mass on the QCM surface in dry conditions, proportionality of the frequency change to the mass deposited retained in liquids, although the sensitivity of this dependency is about one half [46].

**Figure 7:**
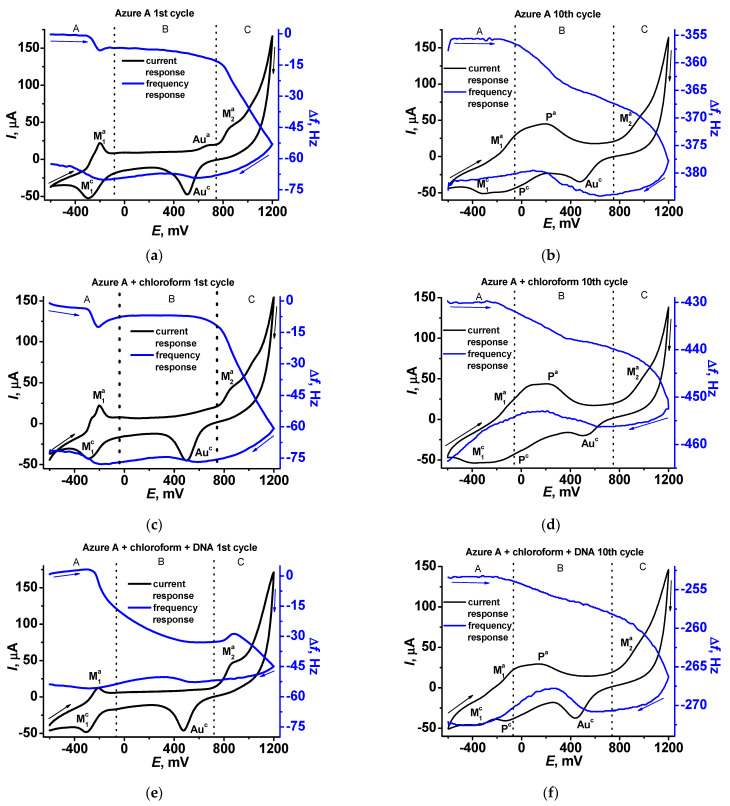
Cyclic voltammograms (black) and sensograms (blue) recorded on the QCM chip in 0.1 M phosphate buffer containing 0.1 M NaNO_3_, pH = 7.0 (**a**,**b**), in the presence of chloroform (**c**,**d**), and in the presence of chloroform and 0.2 mg/mL DNA (**e**,**f**).

In Figure 7, cyclic voltammograms and sensograms are presented for the first (Figure 7a,c,e) and tenth (Figure 7b,d,f) cycles of the potential scanning. In the first scan, well-resolved peaks of the monomeric form (anodic and cathodic peaks M in the A area of the potentials) are present on the cyclic voltammogram together with an irreversible anodic peak initiating polymerization in the C area. Sensograms indicate adsorption of the oxidized form and desorption of the reduced forms of the dye in the A area, which correspond to appropriate shifts of the frequency. In the B area, insignificant changes of the resonance frequency can be found in the absence of DNA, except for a small decrease related to the gold oxidation. In the presence of the DNA, the mass deposited in the B area increases due to electrostatic accumulation of the DNA molecules. In the C area, frequency significantly decreases due to formation of the polymeric form of the dye. It should be noted that DNA adsorbed in the B area negatively affects the polymer deposition due to partial blocking of the electrode surface. The morphology of the sensograms exert a negative trend due to continuing accumulation of the polymer film to the tenth potential scan (Figure 7b,d,f). On voltammograms recorded at the tenth scan, the signals related to the polymer form of the dye appeared (P signals in the A and B areas), indicating redox activity of the film. Meanwhile, the response of the monomeric forms retains conversion to the waves because of the overlapping signal.

**Figure 8:**
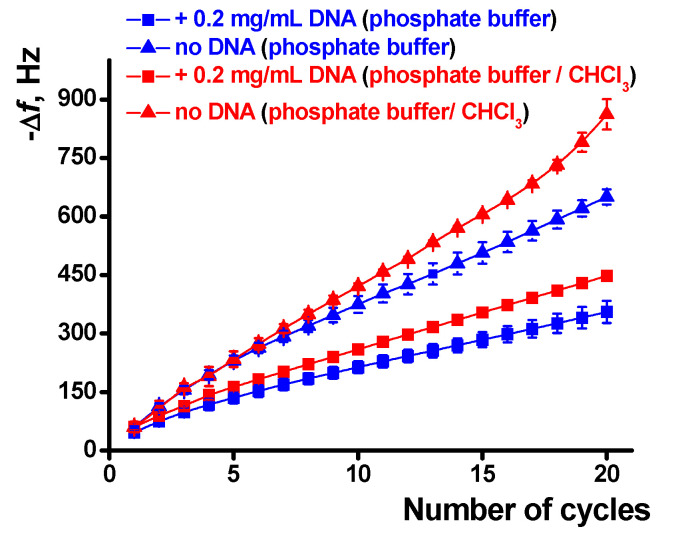
Dependence of the EQCM frequency shift on the number of potential cycles during the poly(Azure A) electropolymerization, average from six QCM chip measurements.

The influence of chloroform and DNA on the polymerization is quantified in Figure 8. The effect is well pronounced in the whole range of the potential scans and is higher for a large number of cycles and the experiments with the DNA addition. The results obtained are well reproducible and show deviation of the frequency change of about 3.5% for six independent EQCM chips modified with the same set of reagents.

It should also be noted that the concentration of the DNA shown in Figure 7 and Figure 8 corresponds to the maximal DNA influence. Lower quantities added do not significantly alter the EQCM parameters while larger ones result in significantly higher deviation of the signals. The increase in the number of potential scans that follow is complicated by lower reproducibility of the results, very long duration of the experiment, and occasional resonance quenching due to overloading of its surface.

#### 3.3.2. Scanning Electron and Atomic Force Microscopy

**Figure 9:**
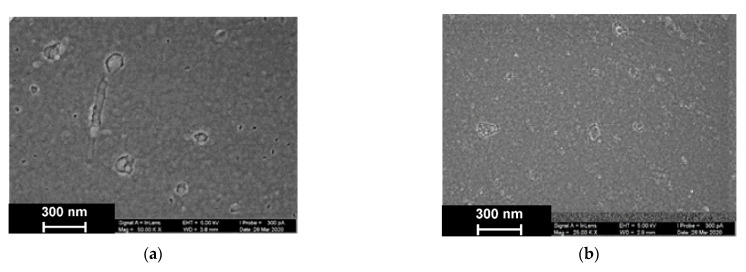

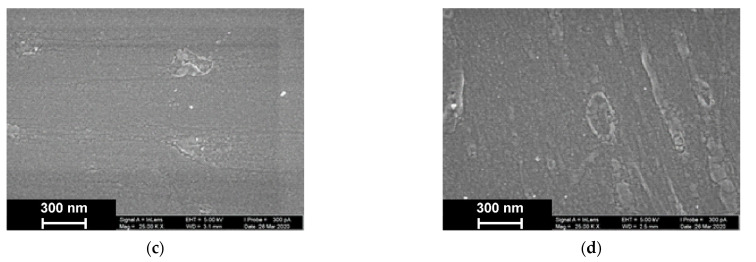
SEM images of the GCE surface covered with Azure A electropolymerized from phosphate buffer (**a**,**c**), in the presence of 0.2 mg/mL DNA (**b**,**d**), and in the absence (**a**,**b**) and presence of CHCl_3_ (**c**,**d**). Twenty cycles of polymerization.

As was established by SEM, electropolymerization of Azure A results in formation of a uniform film with cellular pore structure and roundish defects appearing as dimples with an average diameter of about 40 nm (Figure 9).

The addition of DNA to the monomer solution with no CHCl_3_ makes the layer denser. Additionally, a new kind of defect with spiral or roundish boundaries appeared with an average size of 70 nm. All types of defects with no respect to their size show internal fine crystalline structure. Similar images obtained in phosphate buffer saturated with chloroform indicated formation of the films with a more even surface and smaller domains, some of which were amalgamated in elongated structures.

**Figure 10:**
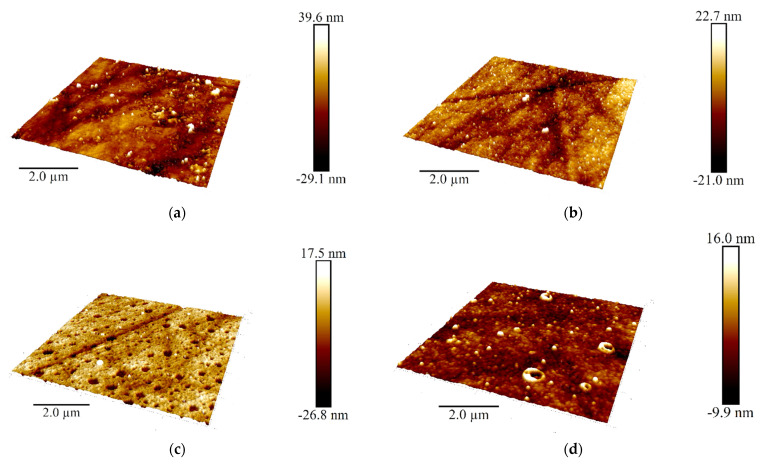

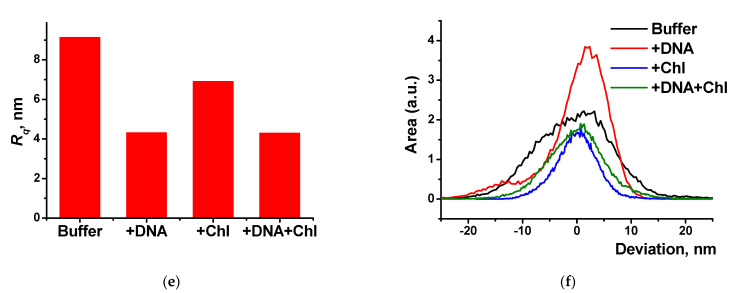
AFM 3D models of the films on the GCE surface covered with Azure A electropolymerized from phosphate buffer (**a**,**c**), in the presence of 0.2 mg/mL DNA (**b**,**d**), and in the absence (**a**,**b**) and in the presence of CHCl_3_ (**c**,**d**); twenty cycles of polymerization. Root mean square values (**e**) and distribution of heights over the 10 × 10 μm^2^ region for the polymers deposited from the buffer and that with addition of DNA (+DNA), saturated with chloroform (+Chl), and that containing both DNA and chloroform (+DNA + Chl) (**f**).

Additional information on the structure of the poly(Azure A) and poly(Azure A)/Chl films was obtained using AMF (Figure 10).

DNA addition to the monomer solution decreased total roughness of the film surface and height deviation within the scan window. The number of peaks on the surface decreased, but their square became larger. In the presence of chloroform and absence of DNA, holes appeared in the film instead of peaks. Most interesting, they were decorated with a kind of oval embankment so that the height difference was maximal near the pores. Cross lines visible on all the AFM images belong to the scratches formed during mechanical polishing of the glassy carbon sheets used for AFM measurements. Probably, the formation of the holes can be related to the dissolution of a part of Azure A aggregates caused by organic solvent. Combination of electrostatic accumulation with negatively charged DNA and disaggregation of dye molecules resulted in formation of a more complex landscape in the case of simultaneous influence of chloroform and DNA on the electropolymerization stage.

#### 3.3.3. Electrochemical Impedance Measurements

**Figure 11:**
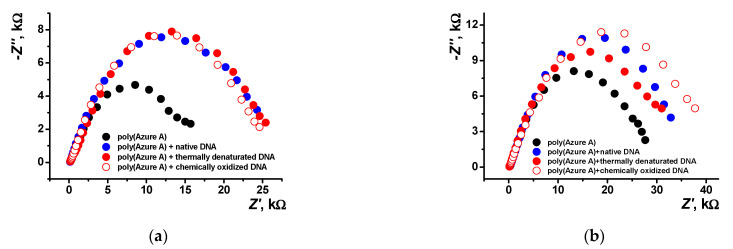
Nyquist diagrams of the impedance spectra recorded on the GCE covered with poly(Azure A) with and without DNA addition for the film–solution interface: (**a**) electropolymerization from aqueous solution of 0.2 mM Azure A in 0.1 M phosphate buffer containing 0.1 M NaNO_3_ and 0.2 mg/mL DNA; (**b**) saturated with chloroform. Frequency range from 100 kHz to 0.04 Hz, amplitude of the applied sine potential 5 mV, pH = 7.0, and twenty cycles.

EIS is a powerful tool for electrochemical measurements that provides valuable information on the surface layer assembly and its influence on the electron transfer at the electrode interface. In this work, EIS measurements were made in an equimolar mixture of 0.01 M [Fe(CN)_6_]^3−^ and [Fe(CN)_6_]^4–^ ions as redox probe. Three types of DNA, i.e., native DNA, DNA chemically oxidized by the Cu^2+^/H_2_O_2_ mixture, and thermally denatured DNA, were tested. Mechanism and conditions for the model DNA damage were established elsewhere [15,47,48]. Figure 11 shows the Nyquist diagrams obtained with the layers obtained in various conditions. Bode diagram (frequency dependence of the phase angle) is presented in Figure S5 (Supplementary Materials)  for various polymer coatings.

As seen in Figure 11b, the introduction of damaged DNA molecules in the growing polymer film increases the radius of the high frequency semicircle. This makes it possible to recognize DNA damaging factors in real sample assay.

**Figure 12:**
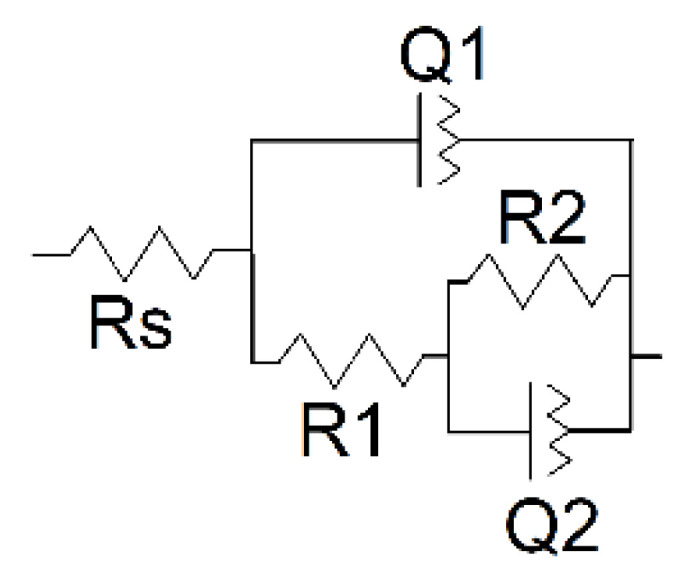
Equivalent circuit for the assessment of EIS parameters.

Interpretation of the EIS data was made using the equivalent circuit earlier proposed for the electrode coated with the porous film (Figure 12) [49,50].

Here, capacitance of the intact coating is represented by the constant phase element (CPE) Q1. R1 (pore resistance) is related to the ion conducting paths in the film formed in electropolymerization. The interface between the electrolyte in the internal film filling and the electrode is modeled by double-layer capacitance (CPE Q2) in parallel with a kinetically controlled charge-transfer reaction (R2). The exponent of the constant phase element (*n*) has been assessed from the Equation (2)

(2) $$Z=\frac{1}{{\left(j\omega \right)}^{n}Q}$$

where *Z* is impedance, *Q* is the CPE, *ω* is angular frequency of a sinusoidal signal, and *j*^2^ = −1. The factor *n* is an adjustable parameter. When *n* differs from 1, the behavior of the system is mostly attributed to the surface heterogeneity [51]. When *n* = 1, the CPE represents an ideal capacitor. Here, *n*1 and *n*2 are related to the *Q*1 and *Q*2 CPE in equivalent circuit presented in Figure 12. The results of the EIS data fitting are presented in Table 1.

Electropolymerization of Azure A in the presence of chloroform shows a lower *n* factor indicating higher porosity of the film. This coincides well with the AFM data. Deposition of the polymer in the presence of DNA makes the film smoother due to partial filling of the pores with the biopolymer molecules. As a result, factor *n* increases together with the R2 values. The charge transfer resistance R1 increases threefold after DNA entrapment. Damaged DNA molecules, being more flexible, decrease this parameter but do not significantly alter R2 values. The effect is less pronounced for the coating obtained in the presence of organic solvent because of higher pore dimensions and lower influence of the DNA molecules on the EIS parameters. Observation changes in the resistance makes it possible to distinguish DNA damaging factors.

## 4. Discussion

The results obtained showed significance of chloroform for the performance of the DNA sensor based on the Azure A polymerization products. Although the saturated buffer solution contained very small quantities of chloroform (0.8%), the polymer films obtained showed significant changes in their parameters important for sensing specific DNA reactions. In the presence of chloroform, the morphology of the surface layer was fully changed: instead of small peaks, regular pores were observed in the absence of DNA. Introduction of DNA in the reaction media containing organic solvent also affected the surface film by partial filling of one pore and unusual decoration of other pores with oval reeds. Although the surface was generally flattened, these changes increased adsorption capability of the polymer, as followed from the SEM/AFM experiments. The effect of chloroform can be explained by partial disaggregation of the monomeric dye molecules onto the growing polymer film and by changes in the hydrophilicity of the surface important for the interactions with the DNA molecules. The efficiency of electropolymerization controlled by the mass changes confirmed the positive effect of chloroform on the deposition of the polymeric form of the dye, both in the absence of DNA and together with the entrapped biopolymer. The mechanism of chloroform influence coincides well with the results of EIS assessment of the capacity and charge transfer resistance that reflect charge separation and efficiency of the ferri-/ferrocyanide transfer through the polymeric film. The advantages of possible application of the DNA sensor developed are shown in the example of discrimination of native and thermally and chemically damaged DNA by their influence on the EIS parameters. Being negligible for traditional electropolymerization, they demonstrated remarkable difference in the case of the coating deposited in the presence of chloroform. This offers new opportunities for the design of electrochemical DNA sensors that can be used in extreme media, including organic solvents, and for providing reliable information on the biochemical interactions with DNA implemented in the surface layer.

## Figures and Tables

**Table 1:** EIS parameters obtained for poly(Azure A)–DNA assembling in various conditions of DNA treatment (average ± S.D. for six electrodes).

Coating Content	*n*1	*R*1, Ω	*Q*1, µF	*n*2	*R*2, Ω	*Q*2, µF
poly(Azure A)	0.64 ± 0.02	2119 ± 390	15.1 ± 2.9	0.78 ± 0.07	16,996 ± 2440	2.43 ± 0.71
poly(Azure A)–DNA	0.76 ± 0.04	6022 ± 738	2.00 ± 0.40	0.64 ± 0.05	24,670 ± 4292	7.64 ± 1.46
poly(Azure A)–thermally damaged DNA	0.66 ± 0.05	1436 ± 465	4.79 ± 0.90	0.67 ± 0.04	25,683 ± 2893	5.52 ± 0.98
poly(Azure A)–oxidatively damaged DNA	0.74 ± 0.10	507 ± 77	4.51 ± 1.4	0.67 ± 0.04	24,776 ± 1740	5.29 ± 1.12
poly(Azure A)/Chl	0.56 ± 0.11	1245 ± 343	6.75 ± 1.90	0.69 ± 0.03	20,751 ± 2057	2.47 ± 0.60
poly(Azure A)/Chl–DNA	0.62 ± 0.03	4029 ± 284	7.12 ± 1.63	0.74 ± 0.05	35,641 ± 2131	2.74 ± 1.04
poly(Azure A)/Chl–thermally damaged DNA	0.57 ± 0.08	1264 ± 288	7.14 ± 2.03	0.70 ± 0.05	29,722 ± 1188	4.40 ± 0.79
poly(Azure A)/Chl–oxidatively damaged DNA	0.67 ± 0.04	942 ± 502	4.89 ± 1.0	0.65 ± 0.04	39,354 ± 4420	6.73 ± 0.97

## Data Availability

No data applicable.

## Supplementary Materials

The following are available online at https://www.mdpi.com/article/10.3390/s21092949/s1, Figure S1: The pH dependence of the anodic and cathodic peak currents of the monomer and polymer of poly(Azure A); Figure S2: Cyclic voltammograms recorded on the GCE covered with poly(Azure A) in the presence of dissolved oxygen and after its removal; Figure S3: The pH dependence of the anodic and cathodic peak currents of the monomer and polymer of poly(Azure A) saturated with chloroform; Figure S4: Cyclic voltammograms recorded on GCE in 0.1 M phosphate buffer containing 0.1 M NaNO_3_ and that saturated with chloroform in the presence of 0.2 mM Azure A and 0.2 mg/mL native and thermally denatured and chemically oxidized DNA. Figure S5: The dependence of the phase angle (φ, °) on the potential frequency obtained with the GCE covered with poly(Azure A) in various conditions. Table S1: The pH dependence of the peak potential of the poly(Azure A) film obtained on GCE.
