# Supplementary material for: Electrochemical DNA Sensor Based on Poly(Azure A) Obtained from the Buffer Saturated with Chloroform
Source: Sensors (Basel). 2021 Apr 22;21(9):2949. doi: 10.3390/s21092949 (PMC8122775; doi:10.3390/s21092949)
Supplement: Supplementary file 1 [file sensors-21-02949-s001.zip › sensors-1186911-supplementary.pdf]

Electronic Supporting Information

to the article of A. Porfireva, K. Plastinina, V. Evtugyn, Y. Kuzin and G. Evtugyn

Electrochemical DNA sensor based on poly(Azure A) obtained from the buffer saturated with chloroform

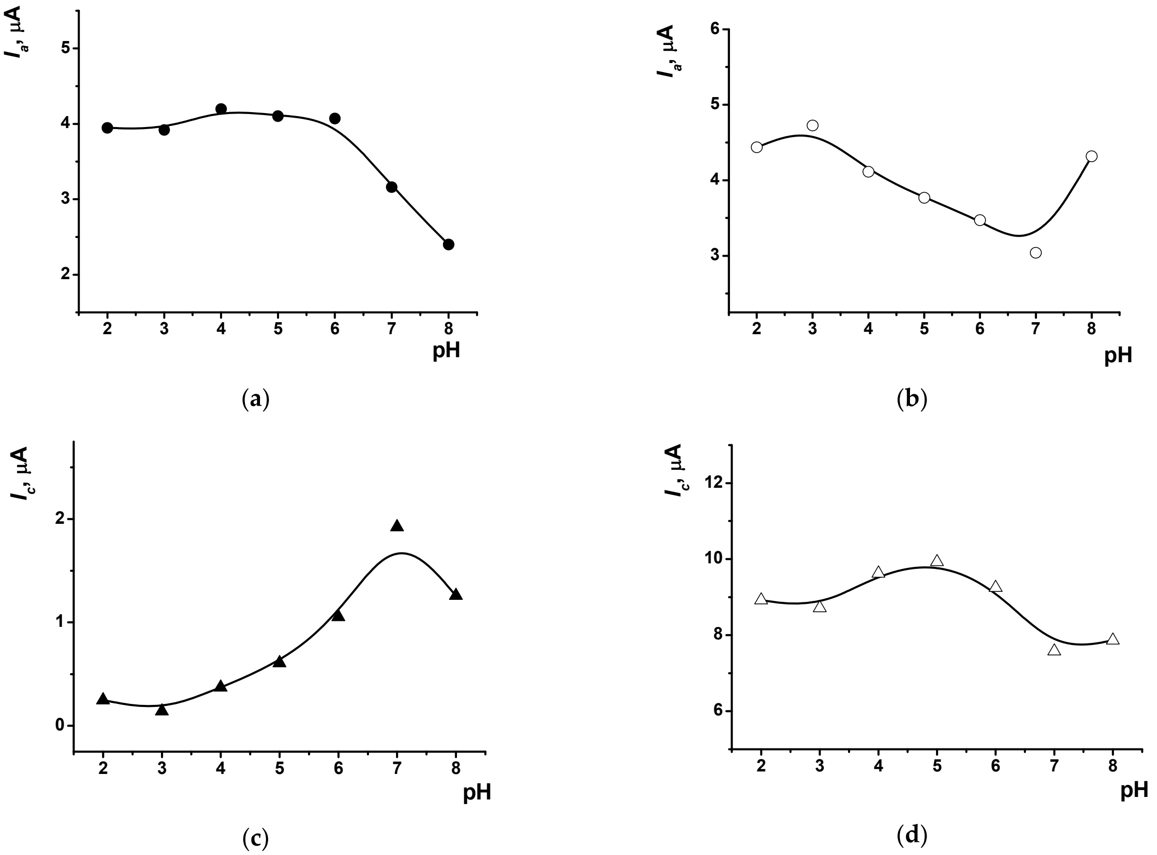

Figure S1. The pH dependence of the anodic peak current of the monomer (**a**) and polymer (**b**), cathodic peak current of the monomer (**c**) and polymer (**d**). Electropolymerization of poly(Azure A) on GCE in phosphate buffer, pH = 7.0, 100 mV/s, twenty cycles.

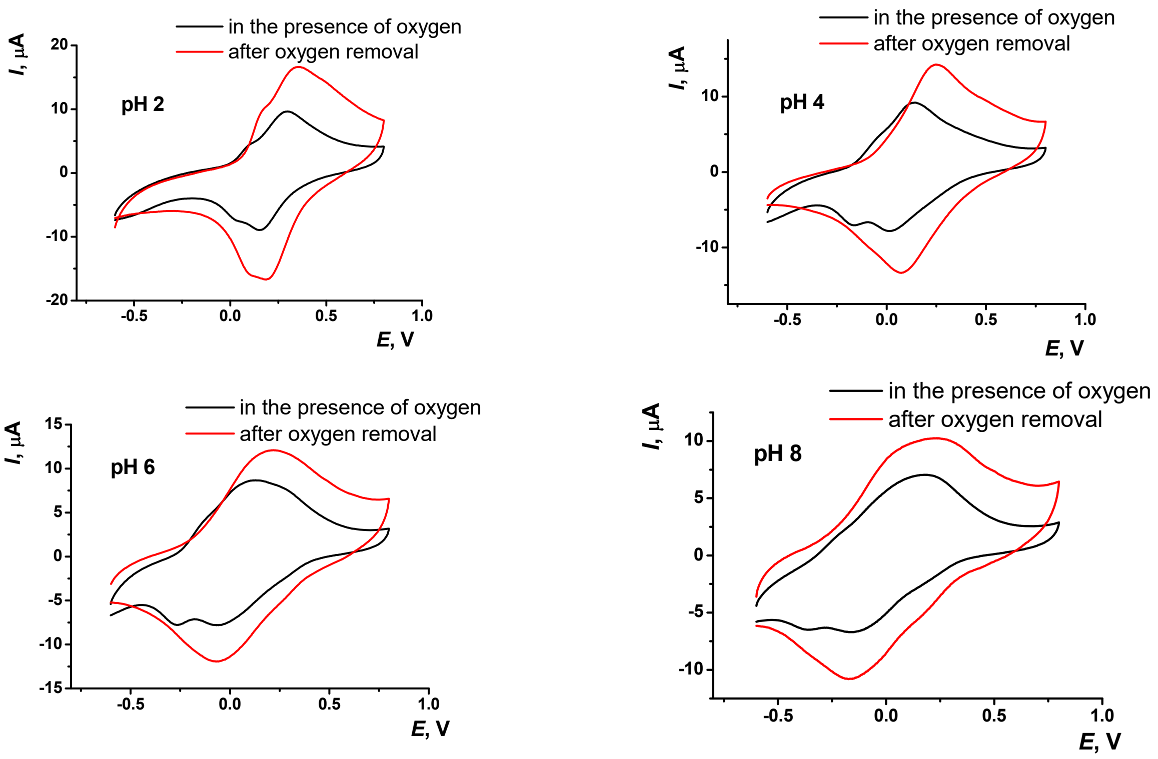

Figure S2. Cyclic voltammograms recorded on the GCE covered with poly(Azure A) in the presence of dissolved oxygen and after its removal. 0.1 phosphate buffer solution, pH 7.0. 100 mV/s, twenty cycles. (**a**) pH 2, (**b**) pH 4, (**c**) pH 6 and (**d**) pH 8.

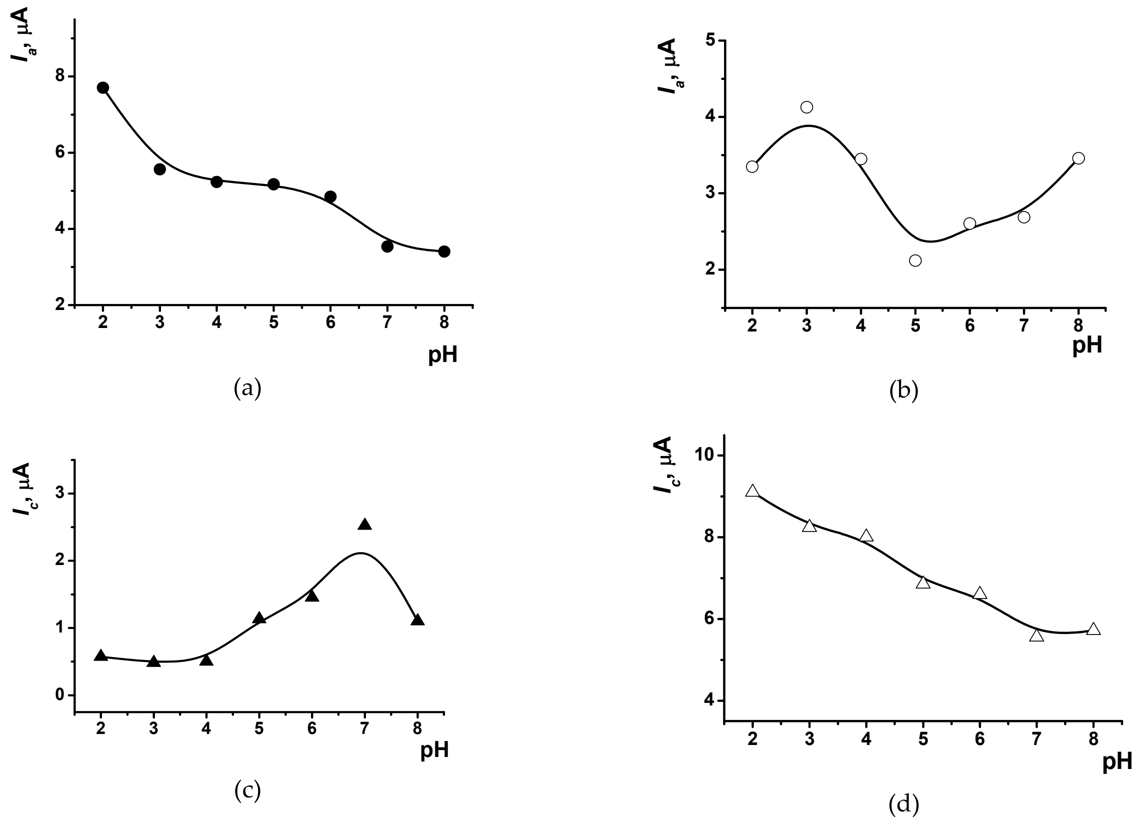

Figure S3. The pH dependence of the anodic peak current of the monomer (**a**) and polymer (**b**), cathodic peak current of the monomer (**c**) and polymer (**d**). Electropolymerization of poly(Azure A) on GCE in phosphate buffer saturated with chloroform, pH = 7.0, 100 mV/s, twenty cycles.

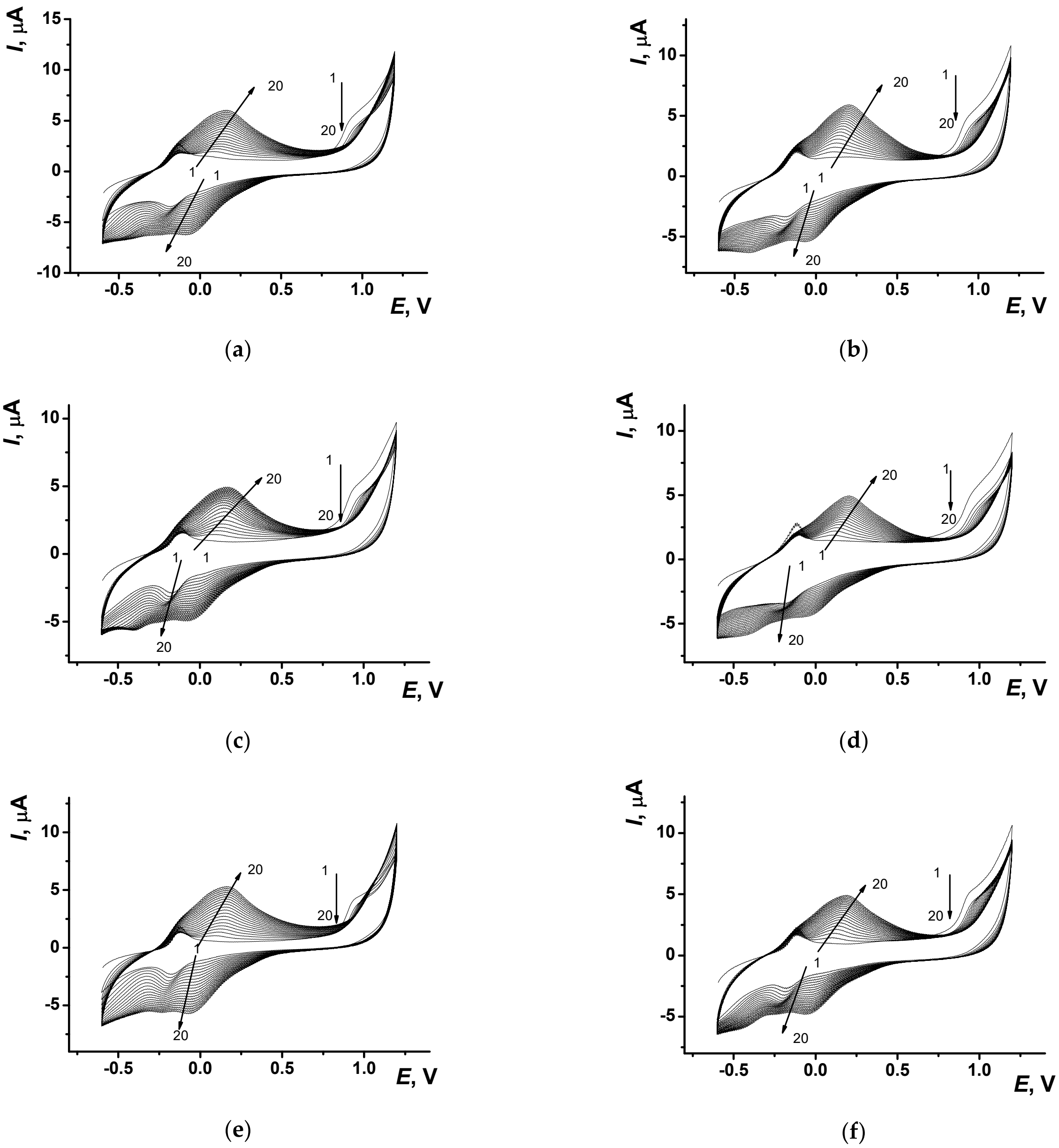

Figure S4. Cyclic voltammograms recorded on GCE in 0.1 M phosphate buffer containing 0.1 M $NaNO_3$ (**a**, **c**, **e**) and that saturated with chloroform (**b**, **d**, **f**) in the presence of 0.2 mM Azure A and 0.2 mg/mL DNA (**a**, **b)** - native DNA, (**c**, **d)** - thermally denaturated DNA, **e**, **f**-chemically oxidized DNA). Twenty cycles of polymerization, pH = 7.0, scan rate 100 mV/s.

Table S1. The pH dependence of the peak potential of the poly(Azure A) film obtained on GCE; *E*, V = a + b·pH. Average from three measurements ± S.D.

Surface layer component	Electrode reaction	a	b	n	R^2
Monomer	Oxidation	0.23 ± 0.01	−0.0573 ± 0.001	7	0.9934
	Reduction	0.23 ± 0.01	−0.083 ± 0.001	6	0.9932
Polymer	Oxidation	0.418 ± 0.001	−0.059 ± 0.001	5	0.9999
	Reduction	0.296 ± 0.012	−0.058 ± 0.003	5	0.9896

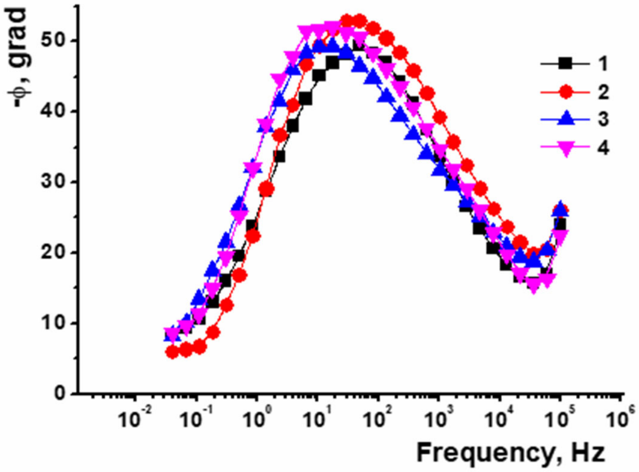

Figure S5. The dependence of the phase angle (φ, °) on the potential frequency obtained with the GCE covered with poly(Azure A) in various conditions. Electropolymerization in 0.1 M phosphate buffer containing 0.1 M $NaNO_3$ and 0.2 mM Azure A (1, 3) and 0.2 mg/mL DNA; (2, 4) in the absence of organic solvent (1, 2) and in the buffer saturated with chloroform (3, 4).
